# Rapidly Progressive Primary Pulmonary Leiomyosarcoma Diagnosed by Endobronchial Ultrasound‐Guided Transbronchial Needle Aspiration: A Case Report

**DOI:** 10.1002/rcr2.70544

**Published:** 2026-03-09

**Authors:** Hayato Yabe, Takumi Nishimaki, Shun Hirabuki, Juri Tsuchimoto, Koutaro Murao, Haruhiko Aisaka

**Affiliations:** ^1^ Department of Respiratory Medicine Japan Community Healthcare Organization Sapporo Hokushin Hospital Sapporo Japan; ^2^ Department of Respiratory Medicine and Allergology Sapporo Medical University School of Medicine Sapporo Japan

**Keywords:** EBUS‐TBNA, primary pulmonary leiomyosarcoma, rare lung tumour

## Abstract

Primary pulmonary leiomyosarcoma (PPL) is a rare malignant tumour with a poor prognosis, particularly in unresectable cases. We report the case of an 82‐year‐old man who was referred for evaluation of an incidental chest abnormality. Chest computed tomography (CT) revealed a nodule in the left upper lobe and enlargement of the 4L lymph node. An initial bronchoscopic biopsy was inconclusive; however, leiomyosarcoma was subsequently diagnosed using endobronchial ultrasound‐guided transbronchial needle aspiration (EBUS‐TBNA) of the lymph node. The patient later developed a solitary brain metastasis detected by brain computed tomography and was treated with Gamma Knife radiosurgery, followed by chemotherapy. Despite treatments, the disease progressed rapidly, and the patient died 4 months after the initial diagnosis. This case highlights the diagnostic value of EBUS‐TBNA in obtaining adequate tissue for definitive diagnosis of rare pulmonary sarcomas and illustrates the aggressive clinical course of PPL with brain metastasis.

## Graphical abstract

Primary pulmonary leiomyosarcoma is a rare malignant tumour that is often difficult to diagnose. We report a case that was successfully diagnosed using endobronchial ultrasound‐guided transbronchial needle aspiration (EBUS‐TBNA) of a mediastinal lymph node. This case highlights the diagnostic value of EBUS‐TBNA in rare pulmonary sarcomas.

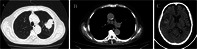

## 1 Introduction

Primary pulmonary leiomyosarcoma (PPL) is an extremely rare malignant tumour that arises from smooth muscle cells in lung parenchyma or bronchial walls [1]. Because of its rarity, the clinical and radiologic features are often indistinguishable from those of more common lung cancers, and a definitive diagnosis usually requires histopathological confirmation. Surgical resection is the treatment of choice for localised disease. However, for patients with unresectable tumours or extrathoracic metastasis, treatment options are limited and the prognosis is poor.

Endobronchial ultrasound‐guided transbronchial needle aspiration (EBUS‐TBNA) is a widely used, minimally invasive, and reliable method for obtaining diagnostic tissue from mediastinal lesions. We report a case of PPL diagnosed by EBUS‐TBNA in an elderly patient, highlighting the diagnostic value of this approach and the aggressive clinical course of the disease.

## 2 Case Report

An 82‐year‐old man was referred for evaluation of an abnormal chest shadow detected on routine imaging. His medical history included hypertension and dyslipidemia, without prior malignancy. His Eastern Cooperative Oncology Group performance status was 1 at presentation. Initial chest CT demonstrated a solitary nodule in the left upper lobe and mild enlargement of the 4L lymph node. Fluorodeoxyglucose positron emission tomography/computed tomography (FDG‐PET/CT) showed intense FDG uptake in the left upper lobe mass (maximum standardised uptake value [SUVmax] 23.5). Absent abdominal or pelvic FDG accumulation excluded extrapulmonary primary tumours.

**FIGURE 1:**
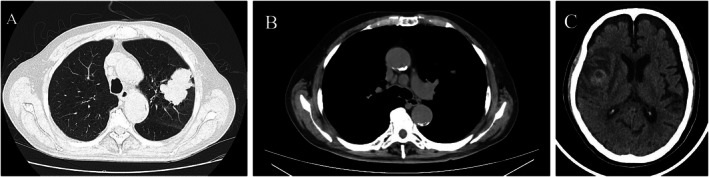
Radiologic findings of primary pulmonary leiomyosarcoma. (A) Axial chest computed tomography (CT) image obtained prior to EBUS‐TBNA showing a solitary mass in the left upper lobe. (B) Axial CT image obtained prior to EBUS‐TBNA showing an enlargement of the 4L lymph node. (C) Non‐contrast brain CT image showing a solitary low‐density lesion with surrounding edema in the right cerebral hemisphere, consistent with brain metastasis.

Bronchoscopic biopsy of the left upper lobe nodule was performed; however, the histologic findings were inconclusive. Follow‐up chest CT performed approximately 3 weeks later prior to EBUS‐TBNA revealed a 50‐mm mass in the left upper lobe and more prominent enlargement of the 4 L lymph node (Figure 1). Given the inconclusive initial biopsy results and progressive nodal enlargement, EBUS‐TBNA of the 4 L lymph node was subsequently performed to obtain adequate tissue for definitive diagnosis and staging, ultimately leading to the histopathological diagnosis of PPL.

**FIGURE 2:**
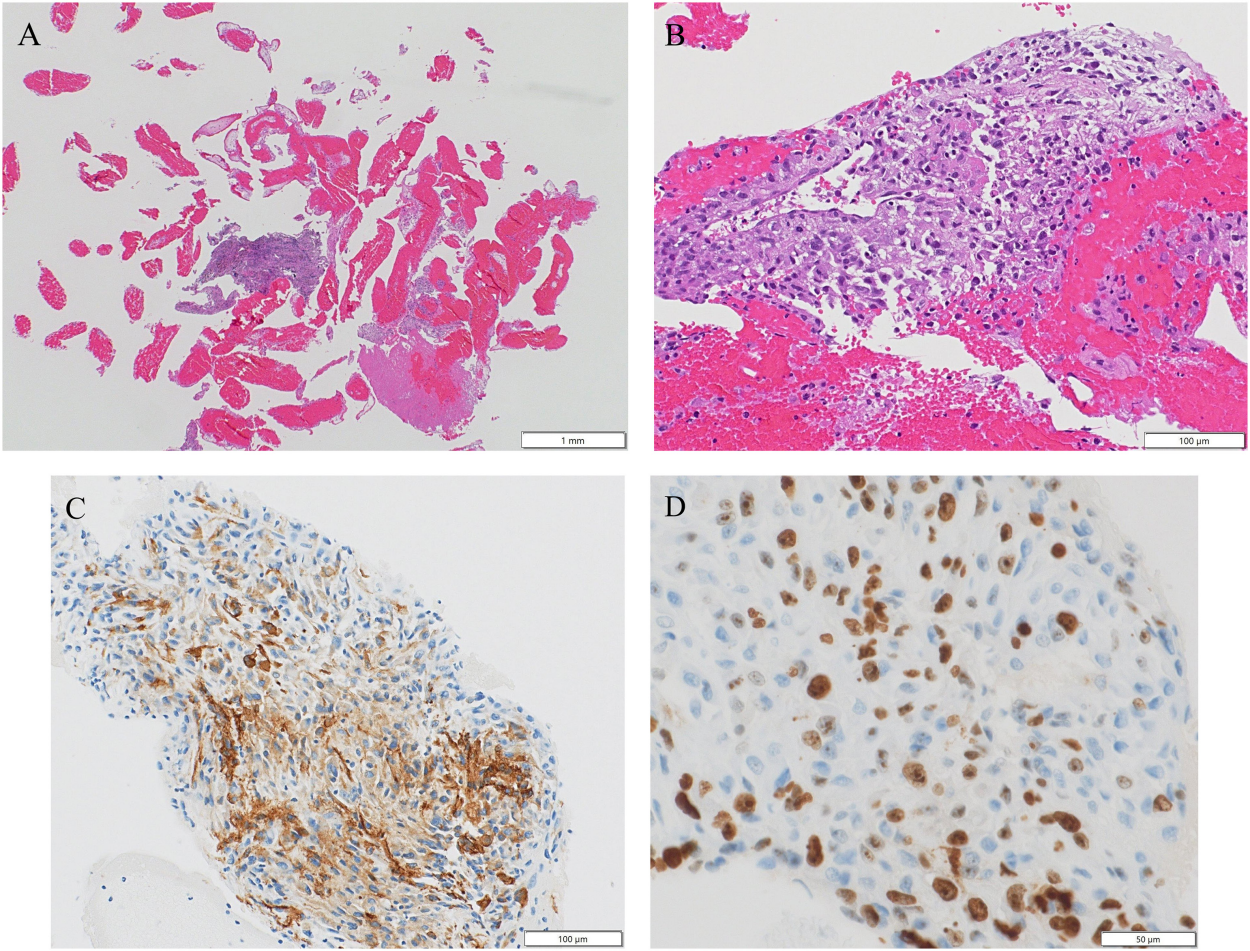
Histopathological and immunohistochemical findings. (A) Haematoxylin and eosin (H&E) staining (×2) showing a solid tumour with focal necrosis. (B) H&E staining (×20) showing proliferation of plasmacytoid to short spindle‐shaped tumour cells with eosinophilic cytoplasm and occasional mitotic figures. (C) Immunohistochemical staining positive for α‐smooth muscle actin (α‐SMA) in tumour cells (×20). (D) Ki‐67 immunostaining showing a high proliferative index of approximately 40% (×40).

Histological examination revealed a solid tumour architecture with focal coagulative necrosis at low magnification and proliferation of plasmacytoid to short spindle‐shaped tumour cells with eosinophilic cytoplasm and a syncytial or loosely cohesive growth pattern. The nuclei were eccentrically located, and occasional mitotic figures were observed. No glandular differentiation or keratinisation was identified. Immunohistochemistry demonstrated positivity for vimentin and α‐smooth muscle actin (α‐SMA). All epithelial markers (CK7, CK5/6, p40, thyroid transcription factor‐1 [TTF‐1], Napsin‐A), lymphoid marker (CD45), melanocytic marker (S‐100), and vascular marker (CD34) markers, as well as desmin and HHF‐35, were negative. The Ki‐67 labelling index was approximately 40% (Figure 2). Programmed death ligand 1 (PD‐L1) expression was < 1%, and next‐generation sequencing using the Oncomine Dx Target Test (ODxTT) did not identify any specific actionable genetic alterations.

Doxorubicin (75 mg/m^2^ every 3 weeks) was administered as first‐line therapy; however, CT after two cycles showed disease progression. Due to the onset of neurological symptoms after two cycles of chemotherapy, head CT was performed, which revealed a solitary metastatic lesion with peritumoral edema in the right cerebrum (Figure 1C). The patient was subsequently referred to another institution for Gamma Knife radiosurgery, which was completed before the initiation of second‐line therapy. Eribulin (1.4 mg/m^2^ on Days 1 and 8 of a 3‐week cycle) was then initiated, but the disease progressed rapidly, and the patient died of respiratory failure 4 months after the initial diagnosis.

## 3 Discussion

PPL is an exceptionally rare malignant tumour of smooth muscle origin, accounting for less than 0.5% of all primary lung malignancies [2]. Its clinical and radiologic features often resemble those of more common lung cancers, which makes accurate diagnosis difficult. In addition, because metastatic leiomyosarcomas from extrapulmonary sites such as the uterus or retroperitoneum, frequently involve the lungs, thorough systemic evaluation including FDG PET/CT is essential to confirm primary pulmonary origin [3]. Although initial systemic evaluation detected no extrathoracic disease, a solitary brain metastasis was identified on head CT after two cycles of doxorubicin, thereby underscoring the potential for rapid metastatic progression. It has been reported that a high SUVmax on FDG PET/CT is associated with a poor prognosis in patients with leiomyosarcoma.

Because these tumours are often firm and may contain necrotic areas, conventional bronchoscopic biopsy may not provide sufficient tissue for a definitive diagnosis. A previous report described a case of PPL diagnosed by EBUS‐TBNA, which was successfully treated with neoadjuvant chemotherapy followed by complete surgical resection [4]. In contrast, our case was unresectable and showed rapid progression despite systemic therapy. In this patient, EBUS‐TBNA of the 4L mediastinal lymph node provided adequate tissue for both histopathological and immunohistochemical evaluation, allowing confirmation of leiomyosarcoma. This case highlights the diagnostic value of minimally invasive sampling techniques in rare pulmonary sarcomas and emphasises the importance of obtaining sufficient tissue to guide appropriate management. Histologically, PPL typically shows spindle to epithelioid tumour cells with smooth muscle differentiation, which is consistent with the findings in this case.

Surgical resection is the mainstay of curative treatment for localised PPL; however, only a minority of patients are eligible because of tumour location, larger tumour size or extrathoracic spread [5, 6]. In this case, surgery was not feasible because of the patient's advanced age and the presence of metastatic lesions. Chemotherapy and Gamma Knife radiosurgery were attempted, but the tumour progressed rapidly, reflecting the highly aggressive nature and poor prognosis of unresectable PPL. This case also highlights the challenges of managing PPL in elderly patients. Although systemic therapy and radiotherapy may provide palliative benefit, treatment outcomes are often limited. Previous reports of prolonged survival in unresectable PPL are rare and typically involve multimodal treatment strategies, including targeted therapies such as pazopanib. In contrast, our patient showed rapid disease progression despite standard therapies, highlighting the heterogeneity of clinical courses in PPL.

In summary, PPL is a rare and aggressive pulmonary malignancy that often presents significant and therapeutic challenges. Adequate tissue sampling using minimally invasive techniques such as EBUS‐TBNA is essential for accurate diagnosis. Although surgical resection remains the only curative option, unresectable cases have a poor prognosis, highlighting the need for novel systemic therapies and a multidisciplinary treatment approach.

## Author Contributions

Hayato Yabe was responsible for patient management, study conception and design, data acquisition and interpretation, and drafting of the manuscript. All other authors contributed to critical revision of the manuscript for important intellectual content and approved the final version.

## Funding

The authors have nothing to report.

## Ethics Statement

This study was conducted following the Declaration of Helsinki.

## Consent

Written informed consent for publication of this manuscript and accompanying images was obtained using the consent form provided by the Journal.

## Conflicts of Interest

The authors declare no conflicts of interest.

## Data Availability

The data that support the findings of this study are available from the corresponding author upon reasonable request.
